# Staged Bilateral Magnetic Resonance‐Guided Focused Ultrasound for Treating Essential Tremor: Systematic Review and Meta‐Analysis

**DOI:** 10.1002/mdc3.70748

**Published:** 2026-08-09

**Authors:** Nima Norbu Sherpa, Andrea Gonzalez Lezana, Gabriela Prilip, Kenji Fukutome, Nathan J. Pertsch, Ruth Reeve, Nadège Bault, Elsa Fouragnan

**Affiliations:** ^1^ Brain Research and Imaging Centre University of Plymouth Plymouth United Kingdom; ^2^ Institute of Psychiatry, Psychology, and Neuroscience King's College London London United Kingdom; ^3^ Instituto de Investigaciones Clínicas Mar del Plata (Institute of Clinical Research Mar del Plata) Buenos Aires Argentina; ^4^ Centro Universitário São Camilo São Paulo Brazil; ^5^ Department of Neurosurgery Osaka Keisatsu Hospital Osaka Japan; ^6^ Department of Neurosurgery Rush University Medical Center Chicago Illinois USA; ^7^ Portsmouth Hospitals University NHS Trust Portsmouth United Kingdom

**Keywords:** bilateral, essential tremor, high intensity focused ultrasound, meta‐analysis, neuromodulation, thalamotomy

## Abstract

**Background:**

Unilateral magnetic resonance guided focused ultrasound (MRgFUS) thalamotomy reduces tremor in essential tremor, yet disability often persists on the untreated side. The added benefit and safety of a staged contralateral lesion remain uncertain.

**Objective:**

The aim was to evaluate the efficacy and safety of staged bilateral MRgFUS thalamotomy for essential tremor.

**Methods:**

We searched MEDLINE, EMBASE, Web of Science, Cochrane CENTRAL, Scopus, ClinicalTrials.gov, and World Health Organization International Clinical Trials Registry Platform from inception to April 12, 2026 (PROSPERO CRD420251027624). Eligible studies were staged bilateral MRgFUS with Clinical Rating Scale for Tremor outcomes and adverse events, and at least 3 months of follow up. Two reviewers extracted data per Preferred Reporting Items for Systematic Reviews and Meta‐Analyses. The primary outcome was the standardized mean difference (SMD) in tremor severity from pre‐ to post‐second procedure. Safety outcomes were pooled 3‐month adverse event proportions using random effects. Risk of bias was assessed with Newcastle‐Ottawa Scale and Risk of Bias in Non‐randomized Studies of Interventions; certainty was graded with Grading of Recommendations Assessment, Development, and Evaluation.

**Results:**

Nine studies were included in the systematic review, of which seven cohorts (n = 149) were included in the meta‐analysis. The pooled SMD was −1.91 (95% confidence intervals, −2.19 to −1.63; *I* ^2^ = 0%), indicating consistent, large‐magnitude tremor reduction across all seven cohorts. Three‐month adverse events included sensory changes (28.8%), gait disturbance (27.5%), dysarthria (22.0%), taste changes (21.7%), dysphagia (18.4%), and ataxia (11.3%). All studies were single‐arm and non‐randomized with moderate to serious risk of bias, and certainty was very low.

**Conclusions:**

Staged bilateral MRgFUS yielded clinically meaningful tremor reduction with predominantly mild adverse events at 3 months. Given very low certainty, findings are hypothesis‐generating and support‐controlled studies with blinded assessment and longer follow‐up.

Essential tremor (ET) is one of the most common movement disorders and frequently causes bilateral action tremor with substantial effects on daily functioning and quality of life.^1^, ^2^ Pharmacological therapy is often inadequate or poorly tolerated, prompting consideration of surgical treatment options.^3^, ^4^ Unilateral magnetic resonance‐guided focused ultrasound (MRgFUS) thalamotomy is a non‐incisional ablative procedure performed with real‐time magnetic resonance (MR) imaging, MR thermometry, and intraoperative clinical testing, and it is now used in routine clinical practice following regulatory approval.^5^, ^6^, ^7^, ^8^, ^9^ MRgFUS is one of several modalities, alongside with deep brain stimulation (DBS), radiofrequency ablation, and γ knife radiosurgery, applied across two main anatomical targets: ventral intermediate nucleus (VIM) of the thalamus and the posterior subthalamic area (PSA), which encompasses the caudal zona incerta and fields of Forel.^10^, ^11^ Growing evidence suggests that PSA targeting may achieve comparable or superior tremor suppression to VIM with a favorable long‐term safety profile.^12^, ^13^

However, many patients remain functionally limited by tremor in the untreated limb,^6^ and unilateral intervention may also be insufficient for some axial symptoms such as head or voice tremor.^14^ Historically, bilateral lesioning raised concerns about dysarthria, gait ataxia, and other speech‐ and balance‐related adverse effects, which limited adoption.^15^ Recent technical developments, including improved imaging, center‐specific targeting refinements, tractography‐informed approaches in some cohorts, and more conservative second‐side lesioning strategies have renewed interest in staged bilateral MRgFUS as a potentially safer approach.^10^, ^11^, ^15^, ^16^, ^17^, ^18^, ^19^, ^20^, ^21^ Centers now use more patient‐specific targeting (eg, diffusion‐tractography informed localization and computerized tomography‐based skull modeling/skull density ratio; with minor second‐side adjustments after confirming no persistent first‐side deficits) and more reliable real‐time monitoring with MR thermometry.^16^, ^22^

Early prospective series and a multicenter open‐label trial suggest that a contralateral staged procedure can provide additional tremor reduction, although persistent mild adverse effects remain an important consideration.^10^, ^11^, ^15^, ^19^, ^20^, ^21^

Despite growing use, important uncertainties remain. Estimates of the benefit of adding a contralateral lesion beyond a successful unilateral procedure, measured on consistent tremor constructs (eg, Clinical Rating Scale for Tremor [CRST] parts A + B), have not been quantitatively synthesized across studies.^18^, ^19^ The frequency and severity of key adverse events particularly dysarthria, dysphagia, paresthesia, ataxia, and gait disturbance vary across centers and time points, complicating guidelines^18^, ^23^ and decision frameworks.^24^, ^25^

We, therefore, conducted a systematic review and meta‐analysis of prospective studies evaluating staged bilateral MRgFUS thalamotomy for the treatment of medicine refractory ET. Our primary objective was to estimate the incremental change in tremor severity post unilateral MRgFUS to the bilateral stages MRgFUS using any validated CRST subscale or combination with standardized mean differences (SMD) applied.^24^ Secondary objectives were to synthesize the prevalence of prespecified adverse events at a harmonized 3‐month time point and to summarize early cognitive outcomes where available.^25^ Given the non‐randomized designs, we appraised risk of bias using Newcastle‐Ottawa Scale (NOS) and Risk of Bias in Non‐randomized Studies (ROBINS) and interpreted certainty accordingly.

## Methods

### Protocol and Registration

This review followed Preferred Reporting Items for Systematic Reviews and Meta‐Analyses (PRISMA) 2020 guidance. The protocol was prospectively registered with PROSPERO (CRD420251027624) before study selection. Any deviations from the protocol are documented in the Supplementary Methods.

### Search Strategy

We systematically searched PubMed/MEDLINE, EMBASE, Cochrane CENTRAL, Web of Science, Scopus, ClinicalTrials.gov, and World Health Organization International Clinical Trials Registry Platform covering all records from inception to April 12, 2026. The following search string was used (adapted per database syntax). No date or language restrictions were applied. Reference lists of all included studies and relevant reviews were also examined to identify additional records. Results were managed in Microsoft Excell and Rayyan, with duplicates removed before screening. We used the following search strategy, “MRgFUS” OR “MR‐guided focused ultrasound” OR “focused ultrasound” OR “High Intensity Focused Ultrasound” OR “HIFU” OR “ultrasound ablation” OR “ultrasonic therapy” OR “ultrasound therapy” AND (“essential tremor” OR “essential tremor”) AND (“bilateral” OR “staged” OR “second procedure” OR “contralateral”). Full search strings for each database are provided in Table S1.

### Selection Criteria

**FIG. 1:**
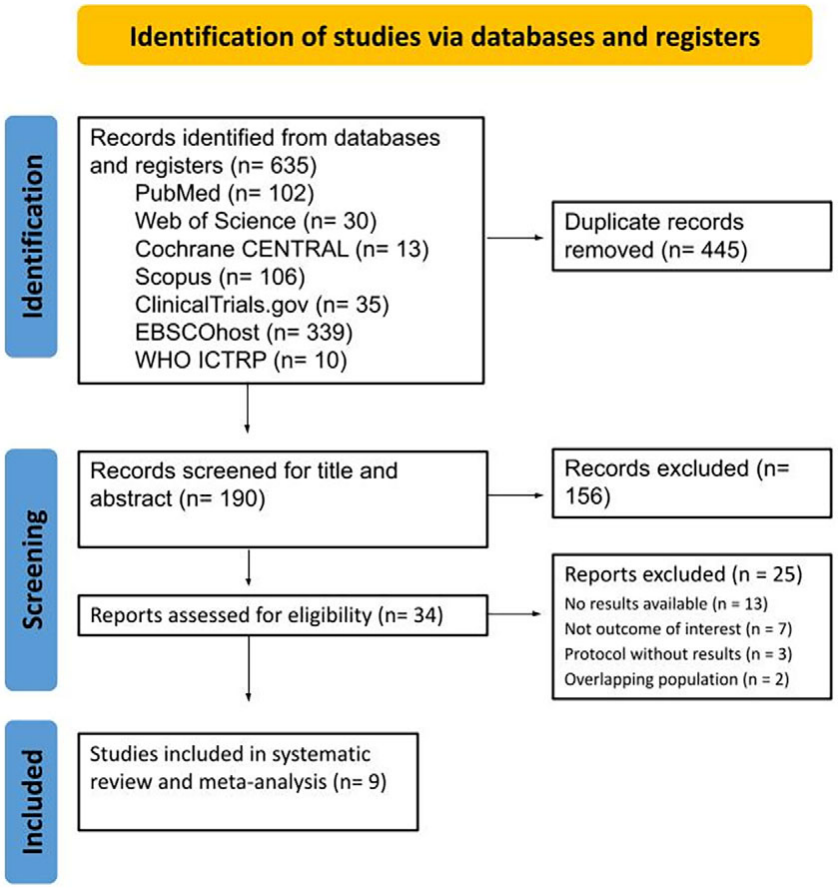
Preferred Reporting Items for Systematic Reviews and Meta‐Analyses (PRISMA) flow diagram of study selection. Flow diagram showing the identification, screening, and inclusion process for studies of staged bilateral magnetic resonance‐guided focused ultrasound (MRgFUS) thalamotomy in essential tremor. WE identified 635 articles across seven databases and registries, we screened for 190 articles after de duplication, 156 articles were excluded based on inclusion and exclusion criteria and finally, nine studies were included in the systematic review, of which seven were included in the meta‐analysis.

Two reviewers (N.N.S. and A.G.) independently screened all studies. Screening was conducted in two stages, stage one was conducted using titles and abstracts, whereas stage two was conducted with full text assessment following inclusion criteria: eligible studies included adult participants with ET undergoing staged bilateral MRgFUS and reporting extractable tremor outcomes after the second procedure and/or prespecified adverse‐event data. Studies reporting any CRST scores or other extractable tremor outcomes were considered eligible, and/or count of prespecified adverse events (dysarthria, gait disturbance, sensory changes, cognitive effects, serious adverse events, and dropouts). Where multiple publications arose from the same center or overlapping cohort, only the most comprehensive non‐overlapping dataset was retained for quantitative synthesis to avoid double‐counting participants. To quantify inter‐rater reliability, Cohen's *κ* statistic was calculated at each stage. Discrepancies at either stage were resolved through discussion, and unresolved disagreements were adjudicated by a third reviewer (G.P.). Where multiple publications reported outcomes from the same cohort, only the most recent report was retained. Accordingly, Iorio‐Morin et al^17^ was superseded by Sarica et al,^19^ which represents the expanded follow‐up of the same prospective trial (NCT04501484). Similarly, Scantlebury et al^21^ was superseded by Boshmaf et al,^26^ which represents the expanded follow‐up of the same Sunnybrook cohort and was, therefore, used in the quantitative synthesis. The study selection process is summarized in the PRISMA flow diagram (Fig. 1).^27^ Where multiple publications reported outcomes from the same cohort, only the most recent report was retained. Accordingly, Iorio‐Morin et al^17^ was superseded by Sarica et al,^19^ which represents the expanded follow‐up of the same prospective trial (NCT04501484).

### Data Extraction

Data extraction was carried out independently by two reviewers (N.N.S. and A.G.L.) using a piloted Excel data‐collection form, with a third reviewer (G.P.) verifying all entries for completeness and accuracy. For each included study, we recorded study identifiers (author, publication year, country, design, and setting), participant characteristics (total sample size, mean age, and sex distribution) and detailed MRgFUS procedural parameters (target nucleus, acoustic energy per sonication in joules, focal‐spot dimensions in millimeters, sonication duration in seconds, number of sonication per session, and inter‐session interval). Efficacy outcomes comprised the pre‐ and post‐second‐session CRST means and standard deviations (SD), together with the timing of the post‐procedure follow‐up. When SDs were not directly reported, we extracted alternative statistics (eg, standard errors or confidence intervals [CIs]) to enable SD imputation. Safety outcomes included the number of participants experiencing each predefined adverse event category such as dysarthria, gait disturbance, sensory changes, cognitive effects, and serious adverse events as well as treatment‐related discontinuations or drop‐outs. When studies used different terms to describe clinically equivalent adverse events, these were unified under a common category (eg, “gait disturbance” and “gait impairment”) to ensure consistency across analyses. In cases of missing or ambiguous data, we contacted corresponding authors by email and documented all responses and remaining data gaps in our extraction log (Supporting Information **S1**).

### Statistical Analysis

All statistical analyses were conducted in R (version ≥4.0)^28^ using the *meta* package.^29^ For our primary efficacy outcome, post‐second procedure values were compared with pre‐second procedure values to estimate the incremental effect of the second‐sided intervention. Because the studies reported tremor outcomes using related, but not fully identical CRST‐based measures (eg, CRST total, CRST A + B, or CRST subcomponents), treatment effects were summarized using SMD with corresponding 95% CIs. A DerSimonian‐Laird random‐effects model^30^ was used to pool effect sizes. Between‐study heterogeneity was assessed using Cochran's *Q* test^29^ and quantified with the inconsistency index (*I* ^2^). Pooled estimates are presented with corresponding 95% CIs. For safety outcomes, proportions of patients experiencing adverse events were stabilized using a Freeman‐Tukey double‐arcsine transformation and pooled using a random‐effects model. Results were back‐transformed to event rates with 95% CIs. To improve consistency across studies, adverse events reported using different, but clinically similar terminology were harmonized into broader categories (eg, sensory changes and taste changes). Events reported in only a single study were described using their corresponding proportions and 95% CIs without formal pooling. Any categories lacking complete raw counts were described narratively. Prespecified subgroup analysis and leave‐one‐out sensitivity analyses were not performed because of the small number of included studies, which would have yielded unstable estimates.

### Study Quality Assessment

Independent evaluation of the methodological quality and risk of bias of each included cohort was conducted by two reviewers (N.N.S. and G.P.) using the NOS^31^ tailored for single‐arm observational studies. The NOS assesses three domains: (1) selection of the study population (representativeness of the cohort, ascertainment of the intervention, demonstration that the outcome was not present at baseline, and adequacy of follow‐up); (2) comparability of participants on key baseline factors (eg, age and baseline tremor severity); and (3) outcome assessment (use of a validated tremor scale, length, and completeness of follow‐up). Each domain awards a maximum number of “stars,” for a total score out of nine. Discrepancies in star ratings were resolved by discussion, with referral to a third reviewer (R.R., N.B., and E.F.) when necessary. Because single‐arm designs cannot fully control for confounding, we supplemented the NOS with a qualitative assessment using the Risk of Bias in Non‐randomized Studies of Interventions (ROBINS‐I) tool.^32^ This assessed potential bias because of confounding, selection of participants, classification of interventions, deviations from intended interventions, missing data, measurement of outcomes, and selective reporting. This quality assessment was also done by two independent reviewers (N.N.S. and G.P.). Overall NOS scores and ROBINS‐I judgments are summarized in Table S1. We conducted our interpretation of pooled effect estimates, with sensitivity analyses excluding any study deemed at high risk of bias. Certainty of evidence was assessed for each outcome using the Grading of Recommendations Assessment, Development, and Evaluation (GRADE) approach. Two reviewers (N.N.S. and G.P.) independently rated the body of evidence across the following domains: risk of bias, inconsistency, indirectness, imprecision, and publication bias. Ratings were classified as high, moderate, low, or very low certainty. Downgrading decisions were made when there were serious concerns in one or more domains (eg, serious risk of bias in all included studies, substantial heterogeneity, small sample sizes, or wide CIs). Disagreements were resolved by consensus or consultation with a third reviewer (R.R., N.B., or E.F.). Disagreements, when they arose, concerned primarily the interpretation of participant selection criteria and the severity of confounding in non‐randomized designs and were resolved through structured discussion in all cases. Discrepancy resolution details are provided in Table S2. Full domain‐level ROBINS‐I assessments are provided in Table S3.

## Results

The initial search yielded 635 results. Overall, nine studies were included in our systematic review, of which seven (n = 149) were included in the meta‐analysis with study completers ranging from (n = 5–45). At the title and abstract stage (n = 88), reviewers agreed on 81 of 88 records (92% observed agreement), yielding a cohen's kappa (*k)*  of 0.83, which represented strong agreement. At the full‐text stage (n = 34), reviewers agreed on 32 of 34 records (94.1% observed agreement), corresponding to a *k* of 0.78, reflecting substantial‐to‐almost perfect agreement.

For efficacy, we used each study's latest available follow‐up (range, 3–12 months). For safety, adverse events were harmonized across all studies at 3 months, which was the common assessment point across cohorts (Table 1).

**TABLE 1:** Study characteristics

Sl no.	Study (country/ setting)	Study type	Participants, n	Age, mean (SD), yr	Male, n (%)	Interval first to second, mean (SD)	Follow‐up schedule	Primary/key outcomes used in this review	Key exclusions/notes
1	Pertsch^38^ 2025 (USA; single center)	Cohort	30	71.2 (8.9)	22 (73.3)	11.6 mo, median (IQR, 9.7–13.8; range, 6.0–21.6)	3, 6, 12, 24, 36 mo after first‐ and second‐side treatment; minimum 3 mo required	CRST part B by arm; directly elicited patient‐reported AEs; satisfaction with second‐side treatment	Near‐consecutive single‐center series; patients with significant first‐side AEs were not eligible, although mild persistent AEs were allowed; symmetric targeting; SDR 0.55 (0.10)
2	Sarica^14^ 2025 (Canada; Toronto Western Hospital)	Cohort	19	69.8 (10.3)	13 (68.4)	17.3 (15.7) mo	Mean follow‐up after second surgery 23.4 (13.3) mo; range, 1–42	Total CRST; lateralized CRST subscores; CTCAE v5 AEs; lesion mapping and connectivity analyses	MRgFUS cohort derived from the Toronto Western prospective phase 2 trial and related records; reviewer advised using this report rather than Iorio‐Morin 2021 in quantitative synthesis because it is the longer overlapping dataset
3	Campins‐Romeu^25^ 2025 (Spain; single center)	Cohort	20	66.5 (9.2)	12 (60.0)	17.8 (7.6) mo	Baseline; FUS1 at 6 mo after first side; FUS2 at 6 mo after second side, with additional AE assessment at 1 mo after second side	CRST A + B, CRST‐C, QUEST, EQ‐5D, Berg Balance Scale, neuropsychological testing, AE grading	Excluded persistent gait, balance, speech, swallowing, or cognitive problems after first side; indirect VIM targeting refined with tractography/DWI; second target placed 1 mm superiorly; SDR 0.56 (0.10)
4	Boshmaf^26^ 2025 (Canada; Sunnybrook, cognition‐focused)	Open label	21	69 (12)	14 (67)	30 (18) mo	Cognitive follow‐up 4.3 (1.8) mo after second side	Comprehensive cognitive battery with group and individual RCI analyses; CRST for the treated hand	Cohort included 11 participants from the earlier safety trial plus 12 standard‐of‐care cases, with 21 in the final cognitive analysis; reviewer advised retaining this report rather than Scantlebury 2023 for the Sunnybrook cohort
5	Kaplitt^7^ 2024 (USA; 7 centers)	Multicenter, open label	45	73.0 (13.9)	44 (86.3)	26.4 (19.2) mo	48 h; 1, 3, 6, 12 mo	CRST parts A + B for treated side at 3 mo; CRST‐C; speech and swallowing assessments; MoCA; structured AE capture	Second side performed at least 9 mo after first; excluded persistent neurological worsening, clinically significant speech/swallowing problems, MoCA <22, prior DBS or stereotactic ablation, and SDR <0.40
6	Fukutome^37^ 2022 (Japan; single center)	Case series	5	57.6	NR	27.8 mo	Baseline; 3 mo after first side; pre‐second side; 3 mo after second side	Total CRST; CRST part C; patient satisfaction; AE reporting	Second operation performed at least 1 year after first; second lesion was placed asymmetrically and more superiorly than the first
7	Martínez‐Fernández^36^ 2021 (Spain/Switzerland; 2 centers)	Open label	9	71.6 (range, 61–80)	5 (55.6)	24 (18) mo	Baseline; FUS1 4 weeks before second thalamotomy; FUS2 6 mo after second thalamotomy	Safety primary; CRST A, B, C, and total; head/voice tremor; neuropsychological testing; VHI	Prior unilateral MRgFUS at least 5 mo earlier; excluded clinically significant permanent AEs after first thalamotomy, prior stereotactic surgery or hemorrhage, unstable cardiac/psychiatric disease, and SDR <0.4
8	Scantlebury^39^ 2023 (Canada; Sunnybrook)	Open label	11	71, range, 52–80	7 (63.6)	At least 12 mo	Baseline and 3–6 mo post‐first side; baseline and up to 6 mo post‐second side, with median 4.4 mo after second side for follow‐up reported in the summary	CRST for each treated hand; systematic AE capture; SARA gait/stance assessment; speech/oro‐motor testing; neuropsychological battery; QUEST	Screened 16 consecutive patients and excluded 5 because of mild cognitive impairment or abnormal gait; overlaps with the later Sunnybrook Boshmaf 2025 cohort and should, therefore, remain descriptive only to avoid double‐counting
9	Iorio‐Morin^12^ 2021 (Canada; Toronto Western, BEST‐FUS phase 2)	Single‐arm, single‐blinded study with blinded external video rating	10	NR	NR	9 mo, median (range, 7–56)	1 and 3 mo after second side	QUEST primary; CRST parts A + B; CRST‐C; CTCAE v5 AE grading; gait and speech assessed by patient and blinded evaluator	Second side only if no persistent gait, speech, or swallowing deficits after first side; overlaps with the later Toronto Western Sarica 2025 dataset and is best retained as a descriptive earlier report rather than pooled separately

*Note*: Interval first→second refers to the time between the first‐ and second‐side MRgFUS procedures.

Abbreviations: SD, standard deviation; yr, year; USA, United States of America; mo, months; IQR, interquartile range; CRST, Clinical Rating Scale for Tremor; AEs, adverse events; SDR, skull density ratio; CTCAE, Common Terminology Criteria for Adverse Events; MRgFUS, magnetic resonance guided focused ultrasound; QUEST, Quality of Life in Essential Tremor Questionnaire; EQ‐5D, EuroQol 5‐Dimension 5‐Level; VIM, ventral intermediate nucleus; DWI, diffusion‐weighted imaging; RCI, reliable change index; MoCA, Montreal Cognitive Assessment; DBS, deep brain stimulation; SARA, Scale for the Assessment and Rating of Ataxia; NR, not reported; QoL, quality of life.

### Tremor Reduction

**FIG. 2:**
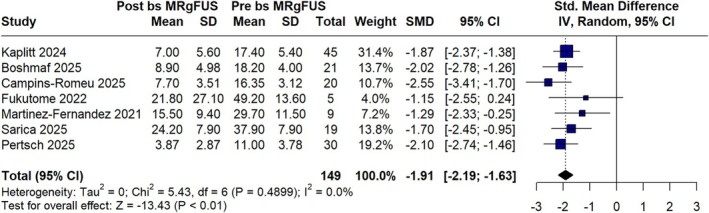
Forest plot of pooled tremor reduction after staged bs MRgFUS. Study‐level and pooled SMD with 95% CI for tremor severity scores from pre‐ to post‐second MRgFUS procedure, analyzed using a DerSimonian‐Laird random‐effects model (IV weighting). Pre‐ and post‐procedure means and SDs are presented for each cohort. Seven studies (n = 149) were included in the pooled analysis. The pooled SMD was −1.91 (95% CI, −2.19 to −1.63), with no evidence of statistical heterogeneity (*I* ^2^ = 0.0%; *τ* ^2^ = 0; *χ* ^2^ = 5.43, *df* = 6, *P* = 0.49). The overall effect was highly significant (*Z* = −13.43, *P* < 0.01). Values to the left of the line of no effect indicate tremor reduction. bs MRgFUS, bilateral MRgFUS; CI, confidence interval; IV, inverse‐variance; SD, standard deviation; SMD, standardized mean difference.

In our quantitative synthesis, staged bilateral MRgFUS was associated with a significant reduction in tremor severity from the pre‐bilateral stage to the post‐bilateral stage, with a pooled SMD of −1.91 (95% CI, −2.19 to −1.63) under a random‐effects model. Between‐study heterogeneity was negligible (*I* ^2^ = 0%; τ^2^ = 0; *Q* = 5.43, degrees of freedom (*df)* = 6, *P* = 0.49), indicating highly consistent effects across the seven included cohorts. Study‐level effects were uniformly in favor of tremor improvement after the second procedure, with SMD ranging from −2.55 to −1.15, and the overall effect remained strongly significant (*Z* = −13.43, *P* < 0.01). The corresponding forest plot presents study‐level means, SDs, inverse‐variance weights, and the pooled estimate across 149 participants (Fig. 2).

### Adverse Events

At 3 months, the most common pooled adverse events were sensory changes (28.8%, 95% CI, 14.9%–44.6%; *I* ^2^ = 65.9%; *k* = 6) (Fig. S1), gait disturbance (27.5%, 95% CI, 13.4%–44.2%; *I* ^2^ = 72.7%; *k* = 5) Fig. S2), dysarthria (22.0%, 95% CI, 5.6%–43.6%; *I* ^2^ = 84.9%; *k* = 6) (Fig. S3), taste changes (21.7%, 95% CI, 14.0%–30.4%; *I* ^2^ = 0%; *k* = 4) (Fig. S4), dysphagia (18.4%, 95% CI 4.5%–38.0%; *I* ^2^ = 79.2%; *k* = 3) (Fig. S5), and ataxia (11.3%, 95% CI, 5.0%–19.4%; *I* ^2^ = 0%; *k* = 3) (Fig. S6). Weakness was uncommon (1.1%, 95% CI, 0.0%–5.7%; *I* ^2^ = 14.1%; *k* = 4) (Fig. S7). Low‐frequency single‐study events that were not pooled included dysmetria, fatigue, decrease in synchronicity, dry mouth, and sialorrhea (Table 2).

**TABLE 2:** Adverse effect analysis

Outcome	Studies, *k*	Pooled prevalence, % (95% CI)	Heterogeneity, *I* ^2^ (%)
Dysarthria	6	22.0 (5.6–43.6)	84.9
Sensory changes	6	28.8 (14.9–44.6)	65.9
Ataxia	3	11.3 (5.0–19.4)	0.0
Taste changes	4	21.7 (14.0–30.4)	0.0
Gait disturbance	5	27.5 (13.4–44.2)	72.7
Dysphagia	3	18.4 (4.5–38.0)	79.2
Weakness	4	1.1 (0.0–5.7)	14.1
Dysmetria	1	1.0 (0.0–5.7)	Not applicable
Fatigue	1	2.0 (0.1–10.7)	Not applicable
Decrease in synchronicity	1	2.0 (0.1–10.7)	Not applicable
Dry mouth	1	2.0 (0.1–10.7)	Not applicable
Sialorrhea	1	2.0 (0.1–10.7)	Not applicable

*Note*: *k* indicates the number of studies included in the pooled analysis. Dashes (−) denote outcomes reported by only one study, for which heterogeneity (*I* ^2^) is not applicable.

Abbreviation: CI, confidence interval.

In the largest multicenter study (Kaplitt et al,^7^ n = 45), 3‐month adverse events included sensory changes nine of 45 (20.0%), dysarthria 8 of 45 (17.8%), ataxia eight of 45 (17.8%), subjective unsteadiness three of 45 (6.7%), taste changes seven of 45 (15.6%), gait disturbance two of 45 (4.4%), hypogeusia four of 45 (8.9%), and dysphagia three of 45 (6.7%). The majority of adverse events at this time point were mild by study definitions, with only isolated moderate events and no procedure‐related neurological events graded severe. In BEST‐FUS phase 2 (Iorio‐Morin et al,^12^ n = 10), dysphagia (2/10), and dysarthria (1/10) were present at 3 months, whereas early gait impairment observed in some participants resolved by 3 months. In the single‐center Spanish cohort (Campins‐Romeu et al,^25^ n = 20), paresthesia (7/20, 35%) and both subjective and objective gait disturbance (3/20 each, 15%) were the most frequent 3‐month findings. All were graded mild and no severe ataxia was reported. Across pooled outcomes, between‐study heterogeneity at 3 months was low for most domains (*I* ^2^ = 0% for dysarthria, taste changes, dysphagia, and ataxia) and moderate for sensory changes and gait disturbance (*I* ^2^ ≈ 51.1%–54.5%), which is compatible with differences in ascertainment (subjective vs. objective gait metrics), targeting nuances, and center‐specific thresholds for proceeding to the second side.

### Cognition and Additional Safety Observations

Cognitive screening at 3 months in the multicenter cohort showed no systematic decline. Boshmaf et al^26^ likewise demonstrated no early cognitive deterioration after the second‐side procedure. Serious harms were rare, beyond an isolated severe urinary tract infection related to peri‐procedural catheter use, no study reported procedure‐related hemorrhage, disabling bilateral neurological deficits, or treatment‐related death.

### Quality Assessment

We assessed study quality using two complementary tools, the NOS and the ROBINS‐I tool for non‐randomized studies. NOS scores ranged from four to nine of nine, indicating moderate to high quality overall. Campins‐Romeu et al^33^ achieved the highest NOS rating (9/9). Kaplitt et al^14^ and Sarica et al^19^ each scored eight of nine. NOS scores for the remaining studies ranged from four to seven, with deductions primarily in the selection and comparability domains.

**FIG. 3:**
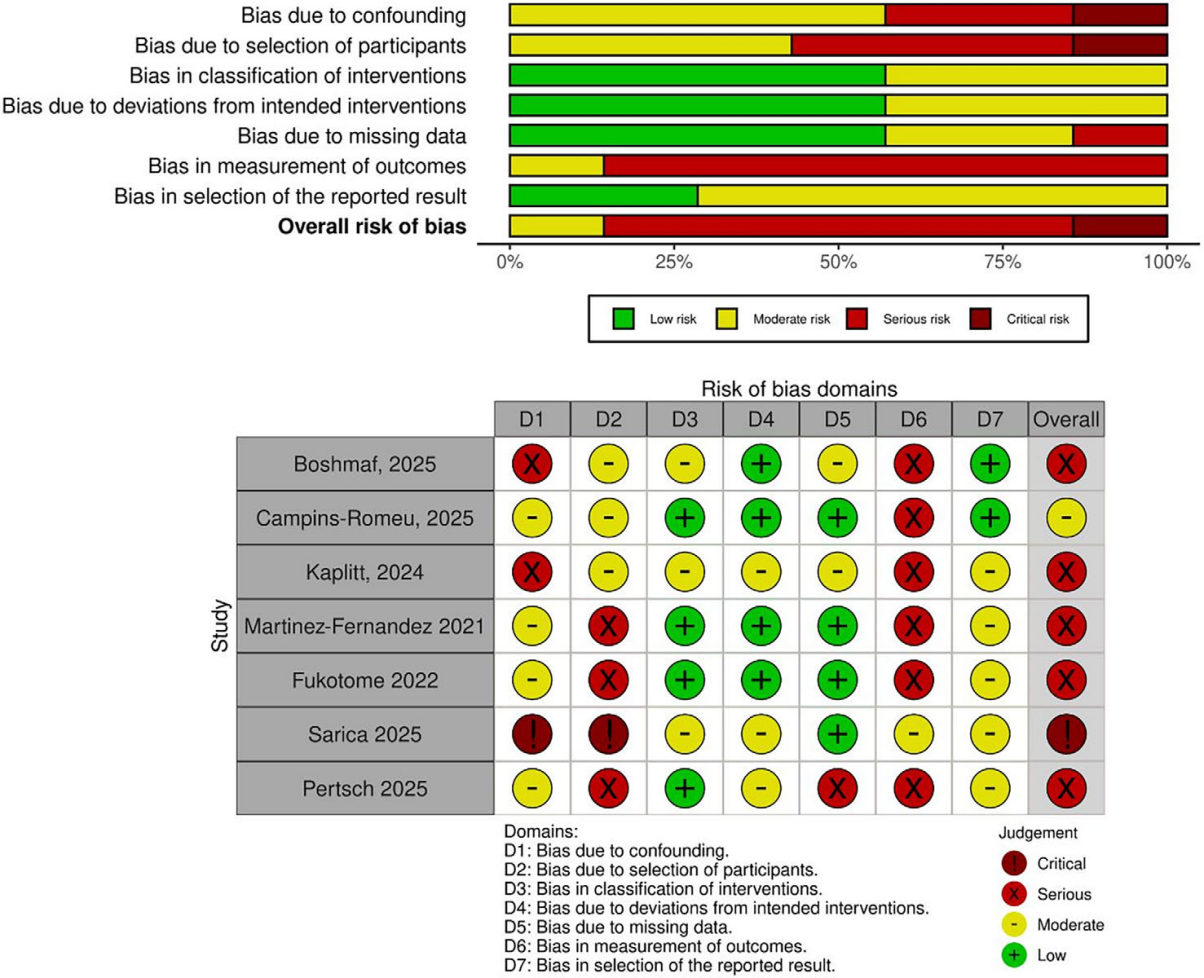
Risk of bias assessment (Risk of Bias in Non‐randomized Studies of Interventions [ROBINS‐I]) across included studies. Risk of bias assessment using ROBINS‐I across seven domains. (A) Summarizes the proportion of studies judged at low (green), moderate (yellow), or serious (red) risk of bias per domain. (B) Shows domain‐level judgments for each study. D1 indicates bias due to confounding; D2, bias due to selection of participants; D3, bias in classification of interventions; D4, bias due to deviations from intended interventions; D5, bias due to missing data; D6, bias in measurement of outcomes; and D7, bias in selection of the reported result.

The ROBINS‐I assessment was conducted across all seven studies included in the meta‐analysis. One study (Campins‐Romeu et al^33^) was judged at moderate overall risk of bias, with the primary concern being unblinded outcome assessment, an inherent limitation of open‐label interventional studies. Five studies (Boshmaf et al,^20^ Kaplitt et al,^14^ Martínez‐Fernández et al,^34^ Fukutome et al,^35^ and Pertsch et al^36^) were rated at serious overall risk of bias, driven by concerns in bias because of confounding or participant selection, reflecting non‐randomized, single‐arm designs with limited adjustment for baseline prognostic factors and bias in measurement of outcomes, as tremor assessors were not blinded to the intervention received. One study (Sarica et al^19^) was rated at critical overall risk of bias, arising from an indirect comparison between a prospectively enrolled MRgFUS cohort and a retrospectively assembled DBS cohort spanning 24 years, with substantial differences in patient selection criteria, baseline characteristics, and inter‐surgery intervals between groups, and no statistical adjustment for these differences. ROBINS‐I domain‐level ratings are summarized in Fig. 3 and Fig. S1.

## Discussion

This systematic review and meta‐analysis of seven studies demonstrates that staged bilateral MRgFUS) thalamotomy confers a clinically meaningful incremental reduction in tremor severity following a successful unilateral procedure. Pooled across seven cohorts^14^, ^19^, ^26^, ^33^, ^34^, ^35^, ^36^ (n = 149), the SMD was −1.91 (95% CI, −2.19 to −1.63; *I* ^2^ = 0%), with all included studies directionally consistent. This uniformity of effect direction, combined with the absence of statistical heterogeneity in the primary estimate, indicates that the transition from the unilateral to the bilateral state produces additional, reliable tremor benefit beyond what unilateral intervention alone achieves.

These findings extend prior reports in three distinct ways. First, they quantify the incremental benefit of staged bilateral treatment using a standardized effect metric that harmonizes across the CRST‐based reporting variations prevalent in the literature, a clinically salient advance for patients who respond to unilateral thalamotomy yet remain functionally limited by tremor on the untreated side.^37^, ^38^ Second, by restricting the adverse event synthesis to the 3‐month horizon, the analysis reduces timing‐related variability and yields tolerability estimates that can be communicated directly in a pre‐procedural counseling setting. Third, integration of a formal quality appraisal using both the NOS and ROBINS‐I framework provides a structured account of the methodological limitations inherent in this evidence base, rather than treating all included cohorts as equivalent.

The adverse event synthesis revealed predominantly mild effects clustered within the domains historically of concern for bilateral thalamic interventions. Moderate to high between‐study heterogeneity was observed for dysarthria (*I* ^2^ = 84.9%), gait disturbance (*I* ^2^ = 72.7%), and dysphagia (*I* ^2^ = 79.2%), most plausibly reflecting variation in targeting strategies, lesion volumes, center‐specific sonication titration approaches, and the methods used to ascertain adverse events (subjective report vs. structured objective assessment). Sensory changes, reported in 28.8% of patients, are particularly noteworthy. VIM thalamotomy is performed in close anatomical proximity to the nucleus ventrocaudalis and medial lemniscus, and the dorsal or superior positional adjustments commonly used for the second‐side procedure may further increase proximity to somatosensory pathways, explaining the elevated frequency of paresthesia relative to unilateral series. Most included studies described intraoperative clinical testing during verification sonications, enabling real‐time assessment of somatosensory effects before ablation; however, reporting of such protocols was not standardized across cohorts, limiting the extent to which intraoperative sensory monitoring can be systematically characterized from the available data.

Targeting strategies varied meaningfully across the included cohorts, and this variability is a plausible contributor to the heterogeneity observed in adverse event profiles. Most centers used conventional VIM coordinate‐based targeting. Campins‐Romeu et al^33^ and Iorio‐Morin et al^17^ both described a 1 mm dorsal shift for the second‐side target to reduce the risk of bilateral adverse effects, whereas Pertsch et al^36^ used symmetric targeting without a standardized directional adjustment. Only Campins‐Romeu et al^33^ explicitly reported diffusion tensor imaging (DTI)‐guided tractography planning. The remaining studies did not describe DTI use. This absence of a consistent targeting standard represents an important methodological gap, and prospective harmonization of second‐side targeting protocols, including the role of connectivity‐informed planning, is a concrete priority for future work. Differences in paresthesia rates and gait‐related outcomes across cohorts likely reflect this targeting variability alongside differences in lesion volume and shape, skull density ratio, operator‐specific titration strategies, and whether gait disturbance was captured subjectively or by objective assessment. Lesion volume was reported in only three of the nine included studies,^17^, ^19^, ^35^ and the metrics used were heterogeneous. Iorio‐Morin et al^12^ reported median volumes of 41 mm^3^ (interquartile range [IQR], 11–73) and 79 mm^3^ (IQR, 57–158) for the first and second procedures, respectively; Fukutome et al^37^ reported means of 64.5 ± 14.1 mm^3^ and 56.8 ± 24.4 mm^3^; and Sarica et al^14^ reported means of 37.9 ± 7.9 mm^3^ and 32.3 ± 13.6 mm^3^. Given the sparse and methodologically inconsistent reporting, a formal comparative analysis of lesion volumes was not feasible, and these figures are presented descriptively only.

Several included studies assessed axial tremor outcomes, specifically head tremor and voice tremor, following staged bilateral MRgFUS thalamotomy, and the available data, while limited, are broadly convergent. Kaplitt et al^7^ observed clinically meaningful improvement in 67% of patients with baseline voice tremor and 71% of those with head tremor. Campins‐Romeu et al^33^ reported improvement of at least one CRST point in 75% and 83% of patients with baseline voice and head tremor, respectively, a finding broadly consistent with Kaplitt et al^7^ and Martínez‐Fernández et al^36^ described an approximately 66% reduction in both voice and head tremor scores following the second procedure, with bilateral intervention achieving gains not observed after unilateral thalamotomy alone. Sarica et al^19^ reported 68% improvement in axial CRST subscores across both bilateral MRgFUS and bilateral DBS cohorts, with no significant difference between modalities. Taken together, these findings suggest that staged bilateral MRgFUS may confer additional axial tremor benefit in a meaningful proportion of patients; however, the evidence base remains limited in size and is insufficient to support definitive conclusions regarding the magnitude of axial benefit or its clinical or anatomical predictors. The remaining studies either did not report axial outcomes or presented insufficient data for analysis.

Inter‐study differences in outcome assessment methodology represent an additional, underappreciated source of heterogeneity. Among the seven cohorts included in the meta‐analysis, blinded video‐rated tremor assessment was used in some studies, whereas others relied on open‐label clinical ratings at the treating center. This distinction is captured in the ROBINS‐I assessment under the domain of bias in measurement of outcomes and has direct implications for effect size interpretation, because open‐label ratings are susceptible to performance and detection bias in a population and clinical team aware of the intervention sequence. The limited number of eligible studies and the absence of standardized reporting of assessment methodology precluded a formal subgroup analysis stratified by blinding status. Future studies should prespecify blinded, video‐rated tremor scoring as part of a core outcome set, alongside standardized adverse event collection at prespecified time points, to substantially reduce this source of bias.

Cognitive safety is an important consideration in both ET and bilateral thalamic interventions.^39^, ^40^ Individuals with ET commonly demonstrate executive dysfunction and carry an increased risk of cognitive decline and dementia.^41^ Given that executive functions underpin independence in daily living and overall quality of life, their preservation is of particular clinical importance.^42^ The included studies conducted limited and inconsistent cognitive assessment, and only Campins‐Romeu et al^25^ undertook a comprehensive neuropsychological evaluation. This represents a significant gap in the current evidence base because without structured pre‐ and post‐procedural neuropsychological testing across all included cohorts, it is not possible to draw conclusions about the cognitive impact of staged bilateral MRgFUS, nor to identify patients who may be at heightened risk. Standardized cognitive assessment should be incorporated as a required component of future bilateral MRgFUS study protocols.

An important consideration in interpreting these findings is that patients undergoing staged bilateral MRgFUS represent an inherently pre‐selected population. Across the included studies, eligibility for the second‐side procedure required the absence of clinically significant persistent adverse effects following the first thalamotomy. Patients who developed persistent gait or balance disturbance,^19^, ^26^ clinically significant speech impairment,^14^ or neurological worsening^14^, ^17^ were explicitly excluded from proceeding to the second‐side procedure. Pertsch et al^36^ permitted patients with mild residual adverse effects, but excluded those with significant impairment. This selection pattern is clinically rational; however, it introduces a systematic selection bias that likely inflates the apparent tolerability profile in the pooled estimates, which is the adverse event rates reported in this meta‐analysis reflect outcomes in patients who were already demonstrably low‐risk based on their first‐procedure experience and not in the broader population of patients with medication‐refractory ET. Clinicians should communicate this important contextual caveat when counseling patients.

The optimal inter‐procedural interval for staged bilateral MRgFUS has not been established by prospective comparative evidence. Across the included studies, intervals between the first and second procedures ranged from approximately 5 months^34^ to more than 60 months,^19^ with most cohorts reporting median or mean intervals of 9 to 28 months. The minimum interval currently applied in regulatory practice reflects approval requirements rather than clinical evidence. The United States Food and Drug Administration supplemental approval for staged bilateral MRgFUS (PMA Supplement P150038/S022, December 2022) specifies a minimum of 9 months based on safety data from the pivotal trial, not on systematic comparison of outcomes across different timing windows. Whether a shorter interval would be safe and effective for patients who are progressing well after the first procedure remains an open clinical question. Future prospective studies should evaluate whether variation in inter‐procedural timing influences both the efficacy and the adverse event profile of the second‐side intervention. After successful unilateral MRgFUS, the decision to pursue contralateral treatment involves a different risk calculus than for bilateral DBS.^43^ With DBS, bilateral procedures remain adjustable and stimulation parameters can be titrated as the clinical picture evolves. Second‐side MRgFUS creates a permanent with significant risks including an 11.3% rate of ataxia and a 28.8% rate of sensory changes observed at 3 months in this population.

Virtually all staged bilateral MRgFUS interventions to date have targeted the ventral intermediate nucleus, yet unilateral studies have begun to investigate alternative targets, including the extrathalamic dentatorubrothalamic tract^34^, ^35^ and the cerebellothalamic tract, as well as the posterior subthalamic area. Future staged bilateral investigations should prospectively evaluate whether connectivity‐informed targeting of these tracts yields outcomes comparable with, or potentially superior to, those achieved through conventional VIM‐focused approaches. Achieving this will require combining standardized clinical outcome measures with lesion segmentation, tractography, and connectivity‐informed planning, a methodological infrastructure that only Campins‐Romeu et al^25^ has so far deployed. Establishing such standards across multicenter consortia represents the most actionable next step for the field.

### Limitations

This meta‐analysis has several limitations that bear directly on the interpretation and generalizability of its findings. First, the analysis is restricted to seven studies comprising 149 patients. This small aggregate sample limits the statistical robustness of pooled estimates and reduces power to detect subgroup differences or modifiers of effect. The findings should, therefore, be regarded as preliminary and hypothesis‐generating rather than definitive. Formal quality appraisal using both the NOS and ROBINS‐I revealed moderate to serious risk of bias, particularly in the domains of confounding and participant selection. The consistently high risk of bias in participant selection is not attributable to individual study flaws, but is instead a structural characteristic of this research domain. Inclusion criteria for the second‐side procedure are necessarily selective, which limits external validity and should be acknowledged as an inherent feature of the current evidence base rather than a correctable methodological deficiency.

Second, although *I* ^2^ was 0% for the primary tremor outcome, moderate heterogeneity was present for several adverse event outcomes, driven by differences in patient characteristics, sonication parameters, targeting strategies, and follow‐up durations. Third, the relatively short follow‐up periods across the included studies, ranging from 3 to 12 months, preclude assessment of long‐term durability and the frequency of persistent bilateral effects. It is not known whether the functional gains observed at 3 to 6 months are maintained at 2 years or beyond, nor whether delayed adverse effects emerge at rates differing from those observed acutely. Fourth, the absence of standardized outcome reporting, including variable use of blinded versus open‐label tremor assessment and inconsistent adverse event ascertainment, introduces detection and performance bias that cannot be corrected in a meta‐analytic framework.

Taken together, these limitations underscore the need for larger, prospectively designed studies with extended follow‐up. Where randomized controlled trials are not feasible, emulated target‐trial designs, propensity‐matched registries, or pragmatic stepped‐wedge approaches represent methodologically credible alternatives. Future studies should include prespecified blinded, video‐rated tremor and speech assessment; time‐resolved safety data collection at peri‐procedural, 1‐, 3‐, 6‐, and 12‐month time points; a core outcome set covering tremor severity, functional independence, speech, balance, patient‐reported quality of life, and cognition; follow‐up extending to at least 12 to 24 months; and prospective imaging‐guided optimization incorporating connectivity‐informed targeting. Adopting these standards across multicenter cohorts would substantially reduce the sources of bias that currently limit confidence in the available evidence.

## Conclusions

Staged bilateral MRgFUS thalamotomy was associated with additional tremor reduction beyond the unilateral state and mostly mild adverse events at 3 months. Given the very low certainty and serious risk of bias, these estimates should be viewed as provisional and hypothesis‐generating. Robust comparative studies with blinded outcomes, standardized safety capture, and longer follow‐up are needed to guide patient selection and counseling.

## Author Roles

(1) Research Project: A. Conception, B. Organization, C. Execution; (2) Statistical Analysis: A. Design, B. Execution, C. Review and Critique; (3) Manuscript Preparation: A. Writing of the First Draft, B. Review and Critique.

N.N.S.:1A, 1B, 1C, 2A, 2B, 2C, 3A, 3B.

A.G.L.: 2A, 2B, 2C.

G.P.: 1B, 1C, 2A, 2C.

K.F.: 2C, 3B, 3C.

H.J.P.: 2C, 3B, 3C.

R.R.: 1C, 2A, 2B, 3A, 3B.

N.B.: 1C, 2A, 2B, 2C, 3A 3B.

E.F.: 1A, 1B, 1C, 2A, 2B, 2C, 3A, 3B.

## Disclosure

**Ethical Compliance Statement:** The authors confirm that the approval of an institutional review board was not required for this work, which is a systematic review and meta‐analysis of previously published data with no new human or animal participants. The authors confirm that patient consent was not required for this work. We confirm that we have read the Journal's position on issues involved in ethical publication and affirm that this work is consistent with those guidelines.

**Funding Sources and Conflict of Interest:** No specific funding was received for this work. N.N.S. is supported by the National Institute for Health and Care Research (NIHR; award 306,286). E.F. is funded by a UK Research and Innovation (UKRI) Future Leaders Fellowship (FLF) (MR/Y034368/1), a Biotechnology and Biological Sciences Research Council (BBSRC) grant (BB/Y001494/1), a Neuromod+ grant (EP/W035057/1), and an Advanced Research and Invention Agency (ARIA) grant (SCNI‐PR01‐P15). The authors declare that there are no conflicts of interest relevant to this work. The authors declare no competing interests.

**Financial Disclosures and Conflicts of Interest:** Within the past 12 months, N.N.S. reports NIHR support (award 306286) unrelated to the submitted work. E.F reports support from UKRI FLF (MR/Y034368/1), BBSRC (BB/Y001494/1), Neuromod+ (EP/W035057/1) and ARIA (SCNI‐PR01‐P15), and an advisory role with Attune Neuroscience, all outside the submitted work. KF reports advisory role for Medtronics. All other authors declare that they have no additional disclosures to report. Author disclosures are available in the Supporting Information.

## Supporting information


**Table S1.** Search strategies and number of records retrieved from each database (PubMed/MEDLINE, EMBASE, Cochrane CENTRAL, Web of Science, Scopus, ClinicalTrials.gov, WHO ICTRP).
**Table S2.** Discrepancy resolution summary for Newcastle–Ottawa Scale (NOS) assessments—per‐reviewer star ratings and final consensus scores.
**Table S3.** ROBINS‐I risk of bias assessment—domain‐level judgments for each of the seven studies included in the meta‐analysis.
**Table S4.** Newcastle–Ottawa Scale (NOS) quality assessment ratings for all nine included cohorts (selection, comparability, and outcome domains) and ROBINS‐I overall risk of bias judgments for the seven meta‐analysis studies.
**Figure S1.** Risk of Bias Assessment (ROBINS‐I)—Summary Panel. Panel A: proportion of studies judged at low (green), moderate (yellow), or serious (red) risk per ROBINS‐I domain. Panel B: domain‐level judgments for each of the seven meta‐analysis studies. Domain codes: D1, confounding; D2, participant selection; D3, classification of interventions; D4, deviations from intended interventions; D5, missing data; D6, measurement of outcomes; D7, selection of reported result.
**Figure S1.** Prevalence of sensory changes at 3 months after second‐side MRgFUS. Forest plot of pooled proportion with 95% confidence intervals (Freeman–Tukey double‐arcsine transformation; random‐effects model).
**Figure S2.** Prevalence of gait disturbance at 3 months after second‐side MRgFUS. Forest plot of pooled proportion with 95% confidence intervals (Freeman–Tukey double‐arcsine transformation; random‐effects model).
**Figure S3.** Prevalence of dysarthria at 3 months after second‐side MRgFUS. Forest plot of pooled proportion with 95% confidence intervals (Freeman–Tukey double‐arcsine transformation; random‐effects model).
**Figure S4.** Prevalence of taste changes at 3 months after second‐side MRgFUS. Forest plot of pooled proportion with 95% confidence intervals (Freeman–Tukey double‐arcsine transformation; random‐effects model).
**Figure S5.** Prevalence of dysphagia at 3 months after second‐side MRgFUS. Forest plot of pooled proportion with 95% confidence intervals (Freeman–Tukey double‐arcsine transformation; random‐effects model).
**Figure S6.** Prevalence of ataxia at 3 months after second‐side MRgFUS. Forest plot of pooled proportion with 95% confidence intervals (Freeman–Tukey double‐arcsine transformation; random‐effects model).
**Figure S7.** Prevalence of weakness at 3 months after second‐side MRgFUS. Forest plot of pooled proportion with 95% confidence intervals (Freeman–Tukey double‐arcsine transformation; random‐effects model).

**Data S1.** Supporting Information.

## Data Availability

All extracted data and analytic code used in this review are available from the corresponding author on reasonable request. The protocol is publicly accessible via PROSPERO (CRD420251027624).

## References

[1] Louis ED , Ferreira JJ . How common is the most common adult movement disorder? Update on the worldwide prevalence of essential tremor. Mov Disord 2010;25:534–541. 10.1002/mds.22838. 20175185

[2] Haubenberger D , Hallett M . Essential Tremor. N Engl J Med 2018;378:1802–1810. 10.1056/nejmcp1707928. 29742376

[3] Zhang J , Yan R , Cui Y , Su D , Feng T . Treatment for essential tremor: a systematic review and Bayesian model‐based network meta‐analysis of RCTs. EClinicalMedicine 2024;77:102889. 10.1016/j.eclinm.2024.102889. 39498461 PMC11533039

[4] Tamburin S , Paio F , Bovi T , et al. Magnetic resonance‐guided focused ultrasound unilateral thalamotomy for medically refractory essential tremor: 3‐year follow‐up data. Front Neurol 2024;15:1360035. 10.3389/fneur.2024.1360035. 38737350 PMC11082386

[5] Pan L , Xiong Y , Li Y , Sun B , Li X , Zhou S , Lou X . Consensus for clinical applications of transcranial magnetic resonance‐guided focused ultrasound. Chin Med J 2024;137:2684–2686. 10.1097/cm9.0000000000003308. 39402972 PMC11611238

[6] Krauss J , Upadhyay N , Purrer V , et al. Beyond the cerebello‐thalamo‐cortical tract: remote structural changes after VIM‐MRgFUS in essential tremor. Parkinsonism Relat Disord 2025;132:107318. 10.1016/j.parkreldis.2025.107318. 39913957

[7] Miller WK , Becker KN , Caras AJ , Mansour TR , Mays MT , Rashid M , Schwalb J . Magnetic resonance‐guided focused ultrasound treatment for essential tremor shows sustained efficacy: a meta‐analysis. Neurosurg Rev 2022;45:533–544. 10.1007/s10143-021-01562-w. 33978922

[8] Mortezaei A , Essibayi MA , Eraghi MM , Alizadeh M , Taghlabi KM , Eskandar EN , et al. Magnetic resonance–guided focused ultrasound in the treatment of refractory essential tremor: a systematic review and meta‐analysis. Neurosurg Focus 2024;57:E2‐2. 10.3171/2024.6.focus24326. 39217634

[9] Agrawal M , Garg K , Samala R , Rajan R , Naik V , Singh M . Outcome and complications of MR guided focused ultrasound for essential tremor: a systematic review and meta‐analysis. Front Neurol 2021;12:12. 10.3389/fneur.2021.654711. PMC8137896 34025558

[10] Dallapiazza RF , Lee DJ , De Vloo P , et al. Outcomes from stereotactic surgery for essential tremor. J Neurol Neurosurg Psychiatry 2019;90:474–482. 10.1136/jnnp-2018-318240. 30337440 PMC6581115

[11] Plaha P , Patel NK , Gill SS . Stimulation of the subthalamic region for essential tremor. J Neurosurg 2004;101(1):48–54. 10.3171/jns.2004.101.1.0048. 15255251

[12] Fan H , Bai Y , Yin Z , et al. Which one is the superior target? A comparison and pooled analysis between posterior subthalamic area and ventral intermediate nucleus deep brain stimulation for essential tremor. CNS Neurosci Ther 2022;28(9):1380–1392. 10.1111/cns.13878. 35687507 PMC9344089

[13] Blomstedt Y , Stenmark Persson R , Awad A , et al. 10 years follow‐up of deep brain stimulation in the caudal zona Incerta/posterior subthalamic area for essential tremor. Mov Disord Clin Pract 2023;10:783–793. 10.1002/mdc3.13729. 37205250 PMC10187013

[14] Kaplitt MG , Krishna V , Eisenberg HM , Elias WJ , Ghanouni P , Baltuch GH , et al. Safety and efficacy of staged, bilateral focused ultrasound Thalamotomy in essential tremor. JAMA Neurol 2024;81(9). 10.1001/jamaneurol.2024.2295. PMC11287440 39073822

[15] Alshaikh J , Fishman PS . Revisiting bilateral thalamotomy for tremor. Clin Neurol Neurosurg 2017;158:103–107. 10.1016/j.clineuro.2017.04.025. 28505539

[16] Kim M , Jung NY , Park CK , Chang WS , Jung HH , Chang JW . Comparative evaluation of magnetic resonance‐guided focused ultrasound surgery for essential tremor. Stereotact Funct Neurosurg 2017;95:279–286. 10.1159/000478866. 28810261

[17] Iorio‐Morin C , Yamamoto K , Sarica C , et al. Bilateral focused ultrasound Thalamotomy for essential tremor (BEST‐FUS phase 2 trial). Mov Disord 2021;36:2653–2662. 10.1002/mds.28716. 34288097

[18] Fornari Caprara AL , Rissardo JP , Byroju V . Bilateral MRgFUS for essential tremor and Parkinson's disease: a literature review (P1‐5.021). Neurology 2025;104:3113. 10.1212/wnl.0000000000210801.

[19] Sarica C , Yamamoto K , Iorio‐Morin C , et al. Bilateral focused ultrasound Thalamotomy for essential tremor: clinical outcomes compared to bilateral deep brain stimulation and probabilistic lesion mapping. Mov Disord 2025;40:1265–1278. 10.1002/mds.30221. 40318052 PMC12273620

[20] van der Stouwe AMM , Jameel A , Gedroyc W , Jones B , Charlesworth G , Molloy S , et al. Double lesion MRgFUS thalamotomy for essential tremor: 4.5‐year outcomes and framework for assessing loss of efficacy and tremor progression. Br J Neurosurg 2024;39(6):1–4. 10.1080/02688697.2024.2354282. 39016204

[21] Scantlebury N , Rohringer CR , Rabin JS , et al. Safety of bilateral staged magnetic resonance‐guided focused ultrasound Thalamotomy for essential tremor. Mov Disord Clin Pract 2023;10(10):1559–1561. 10.1002/mdc3.13882. 37868927 PMC10585969

[22] Bruno F , Catalucci A , Varrassi M , et al. Comparative evaluation of tractography‐based direct targeting and atlas‐based indirect targeting of the ventral intermediate (vim) nucleus in MRgFUS thalamotomy. Sci Rep 2021;11:13538. 10.1038/s41598-021-93058-2. 34188190 PMC8241849

[23] Jackson LM , Kaufmann TJ , Lehman VT , Lee KH , Miller KJ , Hassan A , Klassen BT . Clinical characteristics of patients with gait instability after MR‐guided focused ultrasound Thalamotomy. Tremor Other Hyperkinet Mov 2021;11:41. 10.5334/tohm.643. PMC8533649 34721943

[24] Howard SD , Singh S , Macaluso D , Hsu JY , Cajigas I , Qiu L , et al. Correlation of the clinical rating scale for tremor with a global assessment. Clin Neurol Neurosurg 2025;249:108710. 10.1016/j.clineuro.2024.108710. 39729787

[25] Gasca‐Salas C , Guida P , Piredda R , et al. Cognitive safety after unilateral magnetic resonance‐guided focused ultrasound thalamotomy for essential tremor. J Neurol Neurosurg Psychiatry 2019;90:830–831. 10.1136/jnnp-2018-320129. 30850471

[26] Boshmaf SZ , Petersen J , Kumar R , et al. Cognitive outcomes following second‐sided focused ultrasound Thalamotomy for tremor. Mov Disord Clin Pract 2025;12:1385–1390. 10.1002/mdc3.70095. 40265993 PMC12481419

[27] Page MJ , McKenzie JE , Bossuyt PM , Boutron I , Hoffmann TC , Mulrow CD , et al. The PRISMA 2020 statement: An updated guideline for reporting systematic reviews. Br Med J 2021;372. 10.1136/bmj.n71. PMC8005924 33782057

[28] R Core Team . R: A Language and Environment for Statistical Computing. Vienna: R Foundation for Statistical Computing; 2025.

[29] West SL , Gartlehner G , Mansfield AJ , Poole C , Tant E , Lenfestey N , et al. Table 7, Summary of common statistical approaches to test for heterogeneity; 2010. https://www.ncbi.nlm.nih.gov/books/NBK53317/table/ch3.t2/.

[30] DerSimonian R , Laird N . Meta‐analysis in clinical trials. Control Clin Trials 1986;7:177–188. 10.1016/0197-2456(86)90046-2. 3802833

[31] Wells G , Shea B , O'Connell D , Peterson J , Welch V , Losos M , et al. The Newcastle‐Ottawa Scale (NOS) for assessing the quality of nonrandomised studies in meta‐analyses; 2021. https://www.ohri.ca/programs/clinical_epidemiology/oxford.asp.

[32] ROBINS‐I tool . Cochrane methods. Methods; n.d. https://methods.cochrane.org/robins-i.

[33] Campins‐Romeu M , Conde‐Sardón R , Sastre‐Bataller I , et al. Safety and efficacy of bilateral staged focused ultrasound thalamotomy in refractory essential tremor. Brain Commun 2025;7:fcaf168. 10.1093/braincomms/fcaf168. 40351385 PMC12065003

[34] Martínez‐Fernández R , Mahendran S , Pineda‐Pardo JA , et al. Bilateral staged magnetic resonance‐guided focused ultrasound thalamotomy for the treatment of essential tremor: a case series study. J Neurol Neurosurg Psychiatry 2021;92(9):927–931. 10.1136/jnnp-2020-325278. 33906933

[35] Fukutome K , Hirabayashi H , Osakada Y , Kuga Y , Ohnishi H . Bilateral magnetic resonance imaging‐guided focused ultrasound Thalamotomy for essential tremor. Stereotact Funct Neurosurg 2022;100(1):44–52. 10.1159/000518662. 34515233

[36] Pertsch NJ , Sakakura K , Mueller JM , et al. Tremor control and patient reported outcomes after bilateral focused ultrasound Thalamotomy for essential tremor. Mov Disord Clin Pract 2025;13(7). 10.1002/mdc3.70412. PMC13338991 41159306

[37] Mayo‐Wilson E , Li T , Fusco N , et al. Cherry‐picking by trialists and meta‐analysts can drive conclusions about intervention efficacy. J Clin Epidemiol 2017;91:95–110. 10.1016/j.jclinepi.2017.07.014. 28842290

[38] Shiramba A , Lane S , Ray N , et al. Efficacy and safety of magnetic resonance‐guided focused ultrasound Thalamotomy in essential tremor: a systematic review and Metanalysis. Mov Disord 2025;40(6):1020–1033. 10.1002/mds.30188. 40243386 PMC12160963

[39] Idowu MI , Szameitat AJ . Executive function abilities in cognitively healthy young and older adults—a cross‐sectional study. Front Aging Neurosci 2023;15:976915. 10.3389/fnagi.2023.976915. 36845657 PMC9945216

[40] Kain K , Maller J , Barnett Y , Jonker B , Barnett M , D'Souza A , et al.Tremor suppression following treatment with MRgFUS: skull density ratio consistency and degree of posterior dentatorubrothalamic tract lesioning predicts long‐term clinical outcomes in essential tremor. Front Neurol 2023;14:1129430. 10.3389/fneur.2023.1129430. 37181561 PMC10166854

[41] Janicki SC , Cosentino S , Louis ED . The cognitive side of essential tremor: what are the therapeutic implications? Ther Adv Neurol Disord 2013;6:353–368. 10.1177/1756285613489591. 24228071 PMC3825113

[42] Saporito G , Sucapane P , Ornello R , et al. Cognitive outcomes after focused ultrasound thalamotomy for tremor: results from the COGNIFUS (COGNitive in focused UltraSound) study. Parkinsonism Relat Disord 2023;106:105230. 10.1016/j.parkreldis.2022.105230. 36470172

[43] Prakash P , Deuschl G , Ozinga S , et al. Benefits and risks of a staged‐bilateral VIM versus unilateral VIM DBS for essential tremor. Mov Disord Clin Pract 2022;9:775–784. 10.1002/mdc3.13490. 35937489 PMC9346253

