# Supplementary material for: Staged Bilateral Magnetic Resonance‐Guided Focused Ultrasound for Treating Essential Tremor: Systematic Review and Meta‐Analysis
Source: Mov Disord Clin Pract. 2026 Aug 9:10.1002/mdc3.70748. Online ahead of print. doi: 10.1002/mdc3.70748 (PMC13454638; doi:10.1002/mdc3.70748)
Supplement: Supplementary file 2 [file MDC3-9999-0-s001.docx]

**Supplementary Content**

1) **Protocol**
   Efficacy and Safety of Bilateral Staged Magnetic Resonance–Guided Focused Ultrasound for the Treatment of Essential Tremor: A Systematic Review and Meta-analysis of Single-Arm Prospective Studies Sherpa NN*, Lezana AG, Prilip G, Fukutome K, Pertsch N, Reeve R, Bault N, Fouragnan E
2) **PRISMA Checklist**
3) **Tables**

**eTable 4.** Newcastle–Ottawa Quality Assessment Scale (NOS) Ratings for Included Cohorts
**eTable 2.** Discrepancy Resolution Summary of NOS Assessments
**eTable 1.** Search Strategies and Database Results

4) **Figures**
   **Figure S1.** Risk of Bias Assessment (ROBINS-I)
   **eFigure 6.** Prevalence of Ataxia at 3 Months After Second-Side MRgFUS
   **eFigure 3.** Prevalence of Dysarthria at 3 Months After Second-Side MRgFUS
   **eFigure 4.** Prevalence of Taste Changes at 3 Months After Second-Side MRgFUS
   **eFigure 5.** Prevalence of Dysphagia at 3 Months After Second-Side MRgFUS
   **eFigure 2.** Prevalence of Gait Disturbance at 3 Months After Second-Side MRgFUS
   **eFigure 1.** Prevalence of Sensory Changes at 3 Months After Second-Side MRgFUS
   **eFigure 7.** Prevalence of Weakness at 3 Months After Second-Side MRgFUS

**Protocol**

**Efficacy and safety of Bilateral Staged Magnetic Resonance guided Focused Ultrasound for the treatment of Essential Tremor: a systematic review and meta-analysis of single arm prospective studies**

Sherpa NN*, Lezana AG, Prilip G, Fouragnan E.

**Protocol**

*Correspondence to Nima Norbu Sherpa, Centre for Therapeutic Ultrasound, Brain Research and Imaging Centre, University of Plymouth, 7 Derriford Rd, Plymouth PL6 8BU, United Kingdom; [nimanorbu.sherpa@plymouth.ac.uk](mailto:nimanorbu.sherpa@plymouth.ac.uk)

**Aim**

To evaluate the efficacy and safety of Bilateral Staged Magnetic Resonance guided Focused Ultrasound (bs MRgFUS) for the treatment of Essential Tremor.

**Review question**

1. What is the efficacy of bs MRgFUS on change in tremor severity (as measured by CRST scores) in adults with essential tremor?
2. What is the safety and tolerability profile of bs MRgFUS, assessed by adverse events such as dysarthria, gait disturbance, paresthesia, and cognitive effects—following the second procedure, relative to baseline?

**Search Strategy**

We will include all published and unpublished clinical trials and prospective observational cohort studies that evaluated bs MRgFUS in adults with essential tremor. Databases to be searched include PubMed/MEDLINE, EMBASE, Cochrane CENTRAL, Web of Science, Scopus, ClinicalTrials.gov, WHO ICTRP, and OpenGrey. Reference lists of all included studies and relevant reviews will also be screened. No date or language restrictions will be applied.

**Query search string**: ("MRgFUS" OR "MR-guided focused ultrasound" OR "focused ultrasound" OR "LIFU" OR "low-intensity focused ultrasound") AND ("essential tremor"[Mesh] OR "essential tremor") AND ("bilateral" OR "staged" OR "second procedure" OR "contralateral")

**Types of Studies to Be Included/Excluded**

This review will include prospective clinical investigations of bs MRgFUS in essential tremor. Eligible study designs comprise clinical trials (randomized or non randomized) and observational prospective cohort studies. We will exclude case reports, narrative reviews, editorials, conference abstracts without full data, and studies reporting only unilateral or simultaneous bilateral interventions.

**Condition or Domain Being Studied**

Essential tremor, defined clinically by consensus diagnostic criteria (e.g., Movement Disorder Society guidelines) or ICD/DSM classifications where applicable.

**Participants/Population**

We will include studies enrolling adult humans (≥ 18 years), of any sex or ethnicity, diagnosed with essential tremor. Diagnosis must follow published clinical criteria or validated rating scales. Studies enrolling mixed tremor etiologies will be eligible only if essential tremor–specific outcomes are reported separately or constitute ≥ 80 % of the sample. Participants with comorbid neurological conditions (e.g., Parkinson’s disease), will be excluded unless subgroup data for essential tremor are provided.

**Intervention**

The intervention of interest is bs MRgFUS targeting motor-related nuclei (e.g., ventral intermediate nucleus (VIM), posterior subthalamic area (PSA), or equivalent). Eligible studies must deliver two separate unilateral sonications in distinct sessions (“staged” bilateral), with reporting of sonication parameters, including acoustic energy (Joules), focal spot dimensions (mm), sonication duration (seconds), number of sonications per session, and the inter-session interval (days or weeks).

**Main outcome(s)**
Our primary outcome is the mean change in tremor severity, as measured by the Clinical Rating Scale for Tremor (CRST; Part A, B, or total score), from immediately before the latest follow-up after the second-stage MRgFUS procedure. If studies report different CRST versions or scales, we will convert changes to a common metric and pool them as either mean differences (MDs) or standardized mean differences (SMDs) with 95 % confidence intervals.

Secondary outcomes are safety and tolerability metrics following the second sonication: the proportions of patients experiencing any adverse event (AE), serious AEs, and treatment-related discontinuations or dropouts. Continuous secondary measures (for example, quality of life or functional scores) will be pooled as MDs or SMDs, and binary safety outcomes (AE rates) as pooled proportions with 95 % CIs using a Freeman–Tukey double-arcsine transformation.

**Study Selection and Data Extraction**
Two reviewers will independently screen titles and abstracts against our inclusion criteria (adult essential tremor; staged, bilateral MRgFUS; extractable CRST change or AE data). Full texts of potentially eligible studies will then be assessed in duplicate. Disagreements will be resolved by consensus or a third reviewer. We will document this process in a PRISMA flow diagram.

For each included study, two reviewers will extract data into a standardized form, with another reviewer verifying accuracy. Extracted items will include study identifiers (author, year, country), patient demographics (N, age, sex), MRgFUS parameters (target nucleus, acoustic energy, focal spot size, sonication duration, number of sonications, inter-session interval), follow-up timing, baseline and post-second-session CRST means ± SD (or equivalent), and counts for each AE category. We will contact authors to obtain missing numeric details when necessary.

**Risk of Bias Assessment**
Two reviewers will independently assess each study’s quality using the Newcastle–Ottawa Scale for cohort studies. Domains include selection of the cohort, comparability on key baseline factors, and ascertainment of outcomes. Any discrepancies will be resolved through discussion.

**Strategy for data synthesis**
For the primary efficacy outcome, we will extract or compute the mean change in CRST from baseline to post-second MRgFUS and its SD. If only baseline and follow-up SDs are reported, we will impute the change-score SD using a within-study correlation coefficient of r = 0.5 (with sensitivity analyses at r = 0.3 and 0.7). We will pool mean changes using a DerSimonian–Laird random-effects meta-analysis, reporting MDs (or SMDs if scales differ) with 95 % CIs, the I² statistic, τ², and a 95 % prediction interval.

For safety outcomes, we will pool the proportion of patients experiencing each AE category using a random-effects meta-analysis of proportions with Freeman–Tukey transformation, reporting pooled rates and 95 % CIs. All statistical analyses, forest plots, and funnel plots will be performed in R (version ≥ 4.0). Our annotated code and final meta-analytic dataset will be deposited in a publicly accessible repository to ensure full reproducibility.

**Analysis of subgroups or subsets**

To explore potential sources of heterogeneity in efficacy and safety outcomes, we will conduct the following prespecified subgroup analyses within our single-arm MRgFUS cohorts:

**a)** Target Nucleus Vim-only versus Vim then other protocols. **b)** Acoustic Energy per Sonication Lower energy (< 1 500 J) versus higher energy (≥ 1 500 J) per sonication. **c)**. Follow-Up Duration Short-term (≤ 3 months), intermediate (4–6 months), and long-term (> 6 months) assessments after the second sonication. **d)**. Baseline Tremor Severity Lower severity (CRST total ≤ 25) versus higher severity (CRST total > 25). **e)** Study Size Small cohorts (N < 20) versus larger cohorts (N ≥ 20).

For each subgroup, we will pool mean tremor-score changes and adverse-event rates separately using our random-effects models and compare pooled estimates and I² statistics across strata. Where data allow, we will perform meta-regression to formally test whether acoustic energy or follow-up length predicts effect magnitude or safety outcomes.

**Type and method of review**

Systematic review (and meta-analysis)

**Anticipated or actual start date**

20 June 2025

**Anticipated completion date**

05 July 2025

**Funding**

None

**Country**

United Kingdom

**Subject Index Terms**

Essential Tremor; MR-guided Focused Ultrasound; Bilateral Staged Intervention; Tremor Severity; Clinical Rating Scale for Tremor; Adverse Events; Noninvasive Deep Brain Neuromodulation

**2.PRISMA Checklist**

| **Section and Topic** | **Item #** | **Checklist item** | **Location where item is reported** |
| --- | --- | --- | --- |
| **TITLE** | | |  |
| Title | 1 | Identify the report as a systematic review. | Page 1, line 1 |
| **ABSTRACT** | | |  |
| Abstract | 2 | See the PRISMA 2020 for Abstracts checklist. | Page 4, line 44 |
| **INTRODUCTION** | | |  |
| Rationale | 3 | Describe the rationale for the review in the context of existing knowledge. | Page 6, line 92 |
| Objectives | 4 | Provide an explicit statement of the objective(s) or question(s) the review addresses. | Page 6, line |
| **METHODS** | | |  |
| Eligibility criteria | 5 | Specify the inclusion and exclusion criteria for the review and how studies were grouped for the syntheses. | Page 7, 144 |
| Information sources | 6 | Specify all databases, registers, websites, organisations, reference lists and other sources searched or consulted to identify studies. Specify the date when each source was last searched or consulted. | Page 7, 127 |
| Search strategy | 7 | Present the full search strategies for all databases, registers and websites, including any filters and limits used. | Supplements |
| Selection process | 8 | Specify the methods used to decide whether a study met the inclusion criteria of the review, including how many reviewers screened each record and each report retrieved, whether they worked independently, and if applicable, details of automation tools used in the process. | Page 7, line 141 |
| Data collection process | 9 | Specify the methods used to collect data from reports, including how many reviewers collected data from each report, whether they worked independently, any processes for obtaining or confirming data from study investigators, and if applicable, details of automation tools used in the process. | Page 7, line 142 |
| Data items | 10a | List and define all outcomes for which data were sought. Specify whether all results that were compatible with each outcome domain in each study were sought (e.g. for all measures, time points, analyses), and if not, the methods used to decide which results to collect. | Page 8, line 155 |
|  | 10b | List and define all other variables for which data were sought (e.g. participant and intervention characteristics, funding sources). Describe any assumptions made about any missing or unclear information. | Page 8, line 163 |
| Study risk of bias assessment | 11 | Specify the methods used to assess risk of bias in the included studies, including details of the tool(s) used, how many reviewers assessed each study and whether they worked independently, and if applicable, details of automation tools used in the process. | Page 9, line 195 |
| Effect measures | 12 | Specify for each outcome the effect measure(s) (e.g. risk ratio, mean difference) used in the synthesis or presentation of results. | Page 9, line 183 |
| Synthesis methods | 13a | Describe the processes used to decide which studies were eligible for each synthesis (e.g. tabulating the study intervention characteristics and comparing against the planned groups for each synthesis (item #5)). | Page 7, line 142 |
|  | 13b | Describe any methods required to prepare the data for presentation or synthesis, such as handling of missing summary statistics, or data conversions. | Page 8, line 165 |
|  | 13c | Describe any methods used to tabulate or visually display results of individual studies and syntheses. | Page 11, line 236 |
|  | 13d | Describe any methods used to synthesize results and provide a rationale for the choice(s). If meta-analysis was performed, describe the model(s), method(s) to identify the presence and extent of statistical heterogeneity, and software package(s) used. | Page 9, line 162 |
|  | 13e | Describe any methods used to explore possible causes of heterogeneity among study results (e.g. subgroup analysis, meta-regression). | Page 9, line 184 |
|  | 13f | Describe any sensitivity analyses conducted to assess robustness of the synthesized results. | Page 9, line 192 |
| Reporting bias assessment | 14 | Describe any methods used to assess risk of bias due to missing results in a synthesis (arising from reporting biases). | Page 10, line 196 |
| Certainty assessment | 15 | Describe any methods used to assess certainty (or confidence) in the body of evidence for an outcome. | Page 9, line 214 |
| **RESULTS** | | |  |
| Study selection | 16a | Describe the results of the search and selection process, from the number of records identified in the search to the number of studies included in the review, ideally using a flow diagram. | Page 10, line 216 |
|  | 16b | Cite studies that might appear to meet the inclusion criteria, but which were excluded, and explain why they were excluded. | Page 10, line 229 |
| Study characteristics | 17 | Cite each included study and present its characteristics. | Page 18, line 458 |
| Risk of bias in studies | 18 | Present assessments of risk of bias for each included study. | Page 12, line 287 |
| Results of individual studies | 19 | For all outcomes, present, for each study: (a) summary statistics for each group (where appropriate) and (b) an effect estimate and its precision (e.g. confidence/credible interval), ideally using structured tables or plots. | Page 10, 240 |
| Results of syntheses | 20a | For each synthesis, briefly summarise the characteristics and risk of bias among contributing studies. | Page 12, 294 |
|  | 20b | Present results of all statistical syntheses conducted. If meta-analysis was done, present for each the summary estimate and its precision (e.g. confidence/credible interval) and measures of statistical heterogeneity. If comparing groups, describe the direction of the effect. | Page 10, 245 |
|  | 20c | Present results of all investigations of possible causes of heterogeneity among study results. | Page 11, line 239-284 |
|  | 20d | Present results of all sensitivity analyses conducted to assess the robustness of the synthesized results. | N/A |
| Reporting biases | 21 | Present assessments of risk of bias due to missing results (arising from reporting biases) for each synthesis assessed. | Page 15, line 368 |
| Certainty of evidence | 22 | Present assessments of certainty (or confidence) in the body of evidence for each outcome assessed. | Page 9, line 215 |
| **DISCUSSION** | | |  |
| Discussion | 23a | Provide a general interpretation of the results in the context of other evidence. | Page 13, line 301 |
|  | 23b | Discuss any limitations of the evidence included in the review. | Page 15, line 357 |
|  | 23c | Discuss any limitations of the review processes used. | Page 15, line 376 |
|  | 23d | Discuss implications of the results for practice, policy, and future research. | Page 16, line 385 |
| **OTHER INFORMATION** | | |  |
| Registration and protocol | 24a | Provide registration information for the review, including register name and registration number, or state that the review was not registered. | Page 7, line 128 |
|  | 24b | Indicate where the review protocol can be accessed, or state that a protocol was not prepared. | Page 7, line 129 |
|  | 24c | Describe and explain any amendments to information provided at registration or in the protocol. | Page 7, 130 |
| Support | 25 | Describe sources of financial or non-financial support for the review, and the role of the funders or sponsors in the review. | Page 16, 397 |
| Competing interests | 26 | Declare any competing interests of review authors. | Page 16, 400 |
| Availability of data, code and other materials | 27 | Report which of the following are publicly available and where they can be found: template data collection forms; data extracted from included studies; data used for all analyses; analytic code; any other materials used in the review. | Page 16, 406 |

*From:*  Page MJ, McKenzie JE, Bossuyt PM, Boutron I, Hoffmann TC, Mulrow CD, et al. The PRISMA 2020 statement: an updated guideline for reporting systematic reviews. BMJ 2021;372:n71. doi: 10.1136/bmj.n71

**Figure S1.
Risk of BIAS Assessment**

**NEWCASTLE - OTTAWA QUALITY ASSESSMENT SCALE**

**COHORT STUDIES (NOS)**

**eTable 4. Newcastle–Ottawa Quality Assessment Scale (NOS) Ratings for Included Cohorts**

| **Study (Year)** | **Reviewer 1 (GP)** | **Reviewer 2 (NNS)** | **Consensus (0–9)** |
| --- | --- | --- | --- |
| **Kaplitt 2024** | 8/9 | 8/9 | 8/9 |
| **Campins-Romeu 2025** | 9/9 | 9/9 | 9/9 |
| **Boshmaf 2025** | 7/9 | 7/9 | 7/9 |
| **Iorio-Morin 2021** | 7/9 | 7/9 | 7/9 |
| **Martínez-Fernández 2021** | 8/9 | 7/9 | 7/9 |
| **Fukutome 2022** | 7/9 | 6/9 | 6/9 |
| **Scantlebury 2023** | 5/9 | 4/9 | 4/9 |
| **Pertsch 2025** | 7/9 | 6/9 | 6/9 |
| **Sarica 2025** | 8/9 | 8/9 | 8/9 |

**eTable 2. Discrepancy Resolution Summary of NOS Assessments**

| **Study (Year)** | **Reviewer 1** | **Reviewer 2** | **Consensus Total (0–9)** |
| --- | --- | --- | --- |
| **Iorio-Morin 2021** | 8/9 | 7/9 | 7/9 |
| **Kaplitt 2024** | 8/9 | 9/9 | 8/9 |
| **Boshmaf 2025** | 7/9 | 6/9 | 7/9 |
| **Campins-Romeu 2025** | 9/9 | 8/9 | 9/9 |
| **Martínez-Fernández 2021** | 7/9 | 7/9 | 7/9 |
| **Fukutome 2022** | 6/9 | 6/9 | 6/9 |
| **Sarica 2025** | 8/9 | 8/9 | 8/9 |
| **Pertsch 2025** | 6/9 | 6/9 | 6/9 |
| **Scantlebury 2023** | 4/9 | 4/9 | 4/9 |

**Footnote:**
The Newcastle–Ottawa Scale (NOS) evaluates cohort studies across three domains: *Selection* (maximum 4 stars), *Comparability* (maximum 2 stars), and *Outcome* (maximum 3 stars). Higher scores indicate lower risk of bias. Consensus scores were reached through discussion between reviewers GP and NNS, with arbitration by RR, NB, AGL and EF when required.

**eTable 1.** Search Strategies and Database Results
"MRgFUS" OR "MR-guided focused ultrasound" OR "focused ultrasound" OR “High Intensity Focused Ultrasound” OR “HIFU” OR “ultrasound ablation” OR “ultrasonic therapy” OR “ultrasound therapy” AND ("essential tremor" OR "essential tremor") AND ("bilateral" OR "staged" OR "second procedure" OR "contralateral")

| **Database** | **Hits** |
| --- | --- |
| **PubMed** ((("bilateral" OR "staged" OR "second procedure" OR "contralateral")) AND (("essential tremor" OR "essential tremor"))) AND ("MRgFUS" OR "MR-guided focused ultrasound" OR "focused ultrasound" OR "High Intensity Focused Ultrasound" OR "HIFU" OR "ultrasound ablation" OR "ultrasonic therapy" OR "ultrasound therapy") | **N = 102** |
| **Web of Science**  "MRgFUS" OR "MR-guided focused ultrasound" OR "focused ultrasound" OR "High Intensity Focused Ultrasound" OR "HIFU" OR "ultrasound ablation" OR "ultrasonic therapy" OR "ultrasound therapy" (All Fields) AND "essential tremor" OR "essential tremor" (All Fields) AND "bilateral" OR "staged" OR "second procedure" OR "contralateral" (All Fields) | **N = 30** |
| **Cochrane CENTRAL** ("MRgFUS" OR "MR-guided focused ultrasound" OR "focused ultrasound" OR "High Intensity Focused Ultrasound" OR "HIFU" OR "ultrasound ablation" OR "ultrasonic therapy" OR "ultrasound therapy") AND ("essential tremor" OR "essential tremor") AND ("bilateral" OR "staged" OR "second procedure" OR "contralateral"):ti,ab,kw | **N= 13** |
| **Scopus** (TITLE-ABS-KEY("MRgFUS" OR "MR-guided focused ultrasound" OR "focused ultrasound" OR "High Intensity Focused Ultrasound" OR "HIFU" OR "ultrasound ablation" OR "ultrasonic therapy" OR "ultrasound therapy") AND TITLE-ABS-KEY("essential tremor") AND TITLE-ABS-KEY("bilateral" OR "staged" OR "second procedure" OR "contralateral")) | **N = 106** |
| **ClinicalTrials.gov** Essential Tremor \| ("MRgFUS" OR "MR-guided focused ultrasound" OR "focused ultrasound" OR "High Intensity Focused Ultrasound" OR "HIFU" OR "ultrasound ablation" OR "ultrasonic therapy" OR "ultrasound therapy") | **N= 35** |
| **WHO ICTRP** Essential Tremor AND ("MRgFUS" OR "MR-guided focused ultrasound" OR "focused ultrasound" OR "High Intensity Focused Ultrasound" OR "HIFU" OR "ultrasound ablation" OR "ultrasonic therapy" OR "ultrasound therapy") | **N= 10** |
| **EBSCOhost** TX ( "MRgFUS" OR "MR-guided focused ultrasound" OR "focused ultrasound" OR "High Intensity Focused Ultrasound" OR "HIFU" OR "ultrasound ablation" OR "ultrasonic therapy" OR "ultrasound therapy" ) AND TX essential tremor AND TX ( bilateral OR staged OR "second procedure" OR contralateral ) | **N = 339** |
| **Total** | **N = 635** |
| **Duplicates** | **N= 445** |
| **Unique studies to screen** | **N= 190** |

**FIGURES**

**Supplementary Legends**

**eFigure 6. Prevalence of Ataxia at 3 Months After Second-Side MRgFUS**
Forest plot showing study-level and pooled prevalence of ataxia. Diamonds represent pooled prevalence estimates with 95% CIs; horizontal lines represent study-specific 95% CIs. Heterogeneity was low (I² = 0.0%).

**eFigure 3. Prevalence of Dysarthria at 3 Months After Second-Side MRgFUS**
Forest plot showing pooled prevalence of dysarthria. Heterogeneity was substantial (I² = 84.9%).

**eFigure 4. Prevalence of Taste Changes at 3 Months After Second-Side MRgFUS**
Forest plot showing pooled prevalence of taste changes. Heterogeneity was low (I² = 0.0%).

**eFigure 5. Prevalence of Dysphagia at 3 Months After Second-Side MRgFUS**
Forest plot showing pooled prevalence of dysphagia. Heterogeneity was substantial (I² = 79.2%).

**eFigure 2. Prevalence of Gait Disturbance at 3 Months After Second-Side MRgFUS**
 Forest plot showing pooled prevalence of gait disturbance. Heterogeneity was substantial (I² = 72.7%).

**eFigure 1. Prevalence of Sensory Changes at 3 Months After Second-Side MRgFUS**
Forest plot showing pooled prevalence of sensory changes. Heterogeneity was moderate (I² = 65.9%).

**eFigure 7. Prevalence of Weakness at 3 Months After Second-Side MRgFUS**
Forest plot showing pooled prevalence of weakness. Heterogeneity was low (I² = 14.1%).

**eFigure 6. Prevalence of Ataxia at 3 Months After Second-Side MRgFUS**
Forest plot showing study-level and pooled prevalence of ataxia. Diamonds represent pooled prevalence estimates with 95% CIs; horizontal lines represent study-specific 95% CIs. Heterogeneity was low (I² = 0.0%).

**eFigure 3. Prevalence of Dysarthria at 3 Months After Second-Side MRgFUS**
Forest plot showing pooled prevalence of dysarthria. Heterogeneity was substantial (I² = 84.9%).

**eFigure 4. Prevalence of Taste Changes at 3 Months After Second-Side MRgFUS**
Forest plot showing pooled prevalence of taste changes. Heterogeneity was low (I² = 0.0%).

**eFigure 5. Prevalence of Dysphagia at 3 Months After Second-Side MRgFUS**
Forest plot showing pooled prevalence of dysphagia. Heterogeneity was substantial (I² = 79.2%).

**eFigure 2. Prevalence of Gait Disturbance at 3 Months After Second-Side MRgFUS**
 Forest plot showing pooled prevalence of gait disturbance. Heterogeneity was substantial (I² = 72.7%).

**eFigure 1. Prevalence of Sensory Changes at 3 Months After Second-Side MRgFUS**
Forest plot showing pooled prevalence of sensory changes. Heterogeneity was moderate (I² = 65.9%).

**eFigure 7. Prevalence of Weakness at 3 Months After Second-Side MRgFUS**
Forest plot showing pooled prevalence of weakness. Heterogeneity was low (I² = 14.1%).
